# Responses to Hydric Stress in the Seed-Borne Necrotrophic Fungus *Alternaria brassicicola*

**DOI:** 10.3389/fmicb.2019.01969

**Published:** 2019-08-30

**Authors:** Guillaume Quang N’Guyen, Roxane Raulo, Muriel Marchi, Carlos Agustí-Brisach, Beatrice Iacomi, Sandra Pelletier, Jean-Pierre Renou, Nelly Bataillé-Simoneau, Claire Campion, Franck Bastide, Bruno Hamon, Chloé Mouchès, Benoit Porcheron, Remi Lemoine, Anthony Kwasiborski, Philippe Simoneau, Thomas Guillemette

**Affiliations:** ^1^Institut de Recherche en Horticulture et Semences – UMR 1345, INRA, Université d’Angers, Agrocampus-Ouest, SFR 4207 QUASAV, Angers, France; ^2^Université de Lille, INRA, ISA, Université d’Artois, Université du Littoral Côte d’Opale, EA 7394 - ICV - Institut Charles Viollette, Lille, France; ^3^Departamento de Agronomía, ETSIAM, Universidad de Córdoba, Córdoba, Spain; ^4^Department of Plant Sciences, University of Agronomic Sciences and Veterinary Medicine of Bucharest, Bucharest, Romania; ^5^Equipe “Sucres & Echanges Végétaux-Environnement,” UMR CNRS 7267 EBI Ecologie et Biologie des Interactions, Université de Poitiers, Poitiers, France

**Keywords:** hydrophilins, plant pathogenic fungus, seed transmission, dehydration, *Alternaria brassicicola*

## Abstract

*Alternaria brassicicola* is a necrotrophic fungus causing black spot disease and is an economically important seed-borne pathogen of cultivated brassicas. Seed transmission is a crucial component of its parasitic cycle as it promotes long-term survival and dispersal. Recent studies, conducted with the *Arabidopsis thaliana/A. brassicicola* pathosystem, showed that the level of susceptibility of the fungus to water stress strongly influenced its seed transmission ability. In this study, we gained further insights into the mechanisms involved in the seed infection process by analyzing the transcriptomic and metabolomic responses of germinated spores of *A. brassicicola* exposed to water stress. Then, the repertoire of putative hydrophilins, a group of proteins that are assumed to be involved in cellular dehydration tolerance, was established in *A. brassicicola* based on the expression data and additional structural and biochemical criteria. Phenotyping of single deletion mutants deficient for fungal hydrophilin-like proteins showed that they were affected in their transmission to *A. thaliana* seeds, although their aggressiveness on host vegetative tissues remained intact.

## Introduction

The fungus *Alternaria brassicicola* causes black spot disease and is an economically important seed-borne pathogen of Brassicaceae species. This necrotrophic fungus strongly depends on seed transmission process for its long-term survival and dispersal (van den Bosch et al., 2010). However, fungal and plant factors that impact seed transmission are still poorly described. Such knowledge is crucial to propose strategies for improving the seed health, which remains a major issue for seed companies. Recent studies conducted with the *Arabidopsis thaliana*/*A. brassicicola* pathosystem showed that the level of susceptibility of the fungus to water stress strongly influenced its seed transmission ability. For instance, two osmosensitive fungal mutants, defective for the class III Histidine kinase (HK) AbNik1 (Pochon et al., 2013) and the MAP kinase AbHog1 (unpublished result), respectively, were highly jeopardized in their ability to colonize seeds. Consistently, Iacomi-Vasilescu et al. (2008) had previously reported that *A. brassicicola* spontaneous phenylpyrrole resistant mutants with non-functional class III HK were found to be strongly impaired in their ability to infect radish seeds in field conditions, indicating that a functional high osmolarity pathway is required for efficient infection of seeds. Pochon et al. (2013) also showed that two dehydrin-like proteins were required for effective seed colonization by *A. brassicicola*. During colonization of maturing seeds, the fungi are exposed to severe water and osmotic constraints, and their ability to cope with such stresses is probably a key factor that determines their transmission to seeds.

The strategies to withstand low water availability have been mainly investigated in some organisms called anhydrobiotes belonging to kingdoms of bacteria, fungi and plants, which have the ability to resist desiccation, i.e., to survive to nearly total dehydration (Calahan et al., 2011; Dupont et al., 2014). Various mechanical, structural and oxidative constraints are induced during dehydration, which may lead to damage to membranes, proteins and DNA. Studies in the yeast *Saccharomyces cerevisiae* have shown that the protective system is based on a set of constitutive and inducible mechanisms. For instance, the synthesis of polyols occurs to maintain the osmotic balance. In yeast, the major polyol concerned with osmotic adjustment is glycerol and its synthesis, and hence its intracellular accumulation, is under control of the high osmolarity glycerol (HOG) pathway mediated by phosphorylation of the HOG1 MAP kinase. Some other molecules, such as heat shock proteins, hydrophilins or the non-reducing disaccharides trehalose and sucrose have been identified to be synthesized during desiccation and may be involved in preserving native protein structures or stabilizing membranes (Gadd et al., 1987; Garay-Arroyo et al., 2000; Sales et al., 2000; Tapia and Koshland, 2014).

Hydrophilins are a group of proteins that are present in organisms such as plants, fungi and bacteria, and that are biochemically defined as glycine-rich and highly hydrophilic disordered proteins. They are assumed to be involved in cellular dehydration tolerance, even if their specific role is still unclear. In *Sa. cerevisiae*, 12 genes, whose expression were induced in response to water deficit, encode proteins with specific hydrophilin characteristics (Garay-Arroyo et al., 2000). The deletion (or disruption) and overexpression of these genes showed that only some of them contributed to the desiccation tolerance of yeast (Dang and Hincha, 2011; López-Martínez et al., 2012; Rodríguez-Porrata et al., 2012). A first beneficial effect of hydrophilins on yeast viability during water deficit may be due to their antioxidant capacity which minimizes the accumulation of cellular ROS (López-Martínez et al., 2012). Another role of hydrophilins would be the protection of enzymes and the stabilization of membranes during drying (Sales et al., 2000; Goyal et al., 2005; Reyes et al., 2005). In virtue of their structural plasticity, which is due in part to the presence of disordered regions, hydrophilins may act as molecular shields or directly interact with various molecular partners (Tunnacliffe and Wise, 2007).

Except for the yeast hydrophilins, very little information is available on the presence and role of this class of proteins in filamentous fungi. To date, most studies have focused on a particular class of fungal hydrophilins called dehydrins. Dehydrin-encoding genes were first identified in *Tuber borchii* during searches for genes controlling fruiting body maturation or conidial dormancy (Abba’ et al., 2006). Then, Wong Sak Hoi et al. (2011, 2012) identified three dehydrin-encoding genes in *Aspergillus fumigatus*. Two of them were found to be involved in the protection against oxidative, osmotic and pH stress and the third dehydrin regulated freezing tolerance. A farnesol-induced dehydrin-like protein, that contributed to the high tolerance of resting conidia against oxidative and heat stress, was also identified in *Aspergillus nidulans* (Wartenberg et al., 2012). In *A. brassicicola*, three proteins harboring the typical asparagine-proline-arginine (DPR) signature pattern and sharing the characteristic features of fungal dehydrin-like proteins have been identified (Pochon et al., 2013). The expression of these genes was found to be regulated by the AbHog1 mitogen-activated protein kinase (MAPK) pathway. Phenotyping of single deletion mutants showed that dehydrin-like proteins have an impact mainly on oxidative stress tolerance and on conidial survival upon exposure to high and freezing temperatures. Moreover, a double deletion mutant was strongly affected with respect to conidiation and showed a highly compromised pathogenicity with a lower aggressiveness on *Brassica oleracea* leaves and a reduced capacity to be transmitted to *Arabidopsis* seeds via siliques. These results support the importance of dehydrin proteins and, more globally, of hydrophilins, with respect to the ability of *A. brassicicola* to efficiently accomplish key steps of its pathogen life cycle.

To identify new fungal molecular determinants that may be involved in the silique and seed infection process, the present study aimed at analyzing the transcriptomic and metabolomic responses of *A. brassicicola* exposed to water stress. Then, the repertoire of putative hydrophilins in this fungus was established based on expression data and structural and biochemical criteria. Finally, functional approaches were initiated to investigate the role of some of these putative hydrophilins.

## Results

### Application of Water Stresses

Several preliminary tests were conducted to evaluate the level of stress for each treatment applied to fungal cells. Considering that the reference strain is naturally tolerant to water stress (Iacomi-Vasilescu et al., 2008), we additionally analyzed the effect of treatments on three mutant strains (Δ*abnik1*, Δ*abhog1* and Δ*absch9*) derived from the wild-type Abra43 strain, for which we hypothesized a higher sensitivity to water stress. As expected, the Abra43 reference strain exhibited high sorbitol tolerance with only 5% inhibition at a concentration of 1.2 M. At this concentration, a higher susceptibility was observed for the mutant strains (Table 1), confirming that the application of such a concentration indeed constitutes a stress for the fungal cells. In the same way, when a drastic decrease in relative humidity (RH) was applied to the germlings, no significant difference in mortality between the treated and the silica gel-free conditions was observed for the reference strain. On the contrary, the results showed a very high susceptibility of Δ*abnik1* and Δ*abhog1* to the desiccation stress, while the *AbSch9* mutant exhibited an intermediate susceptibility (Table 1).

**TABLE 1:** Susceptibility of *Alternaria brassicicola* wild-type, Δ*Abnik1*,Δ*Abhog1* and Δ*Asch9* strains to sorbitol and desiccation stresses.

**Strains**	**% growth inhibition Sorbitol (1.2 M)**	**Mortality rate after 4 days of desiccation**
WT	5 ± 2	5 ± 2
Δ*Abnik1*	29 ± 4	98 ± 2
Δ*Absch9*	48 ± 5	35 ± 5
Δ*Abhog1*	39 ± 4	98 ± 1

*Each condition was tested in triplicate and the experiments were repeated twice. Values are means of biological repetitions and represent the percentage growth inhibition under stress conditions compared with standard growth conditions.*

Based on these results, we chose to analyze the responses of the fungus when exposed to 1.2 M sorbitol for 0.5 and 2 h and when exposed to desiccation stress for 1 and 4 h. Regarding the latter stress, the chosen times implied that germlings exposed to silica gel beads were subjected to a RH value of 1% while control germlings were harvested at RH values of 70% and 90%, respectively.

In addition, we analyzed other phenotypic characteristics of these mutants: the susceptibility to oxidative stress (exposure to 10 mM H_2_O_2_ and 20 mM menadione), the ability to transmit to the *A. thaliana* seeds and the aggressiveness on cabbage leaves. Δ*abnik1* and Δ*abhog1* mutants were more susceptible to oxidative stress, and, consistently, had reduced ability to colonize *A. thaliana* seeds compared to the wild-type strain (Table 2). The Δ*absch9* mutant was not susceptible to oxidative stress and its seed transmission capacity was not altered. Compared to seed transmission capacity, the ability of leaf infection was not or less dependent on the sensitivity of fungal genotypes to oxidative stress, since Δ*abnik1* and Δ*absch9* strains did not have reduced aggressiveness on cabbage leaves.

**TABLE 2:** Other phenotypic characteristics of kinase mutants considered in this study.

**Fungal strains (origin)**	**Susceptibility to oxidants**		**Seed transmission efficiency**	**Symptom development on leaves**
	**H_2_O_2_ (2.5 mM)**	**Menadione (5 mM)**		
*WT*	22.2 ± 2	34,2 ± 3		
Δ*abNik1* (Dongo et al., 2009)	54.2 ± 5^a^	64.5 ± 6 ^a^	– (Pochon et al., 2012)	0 (Pochon et al., 2012)
Δ*abHog1* (Joubert et al., 2011)	79.5 ± 6 ^a^	84.5 ± 2 ^a^	– (This study)	– (Joubert et al., 2011)
Δ*abSch9* (this study)	11 ± 3 ^a^	21 ± 1 ^a^	0 (This study)	0 (This study)

*Values were obtained in this study and are means of three biological repetitions and represent the percentage growth inhibition under stress conditions compared with standard growth conditions. ^*a*^indicates a significant difference between the mutant and the Abra43 WT parental isolate (Student’s *t*-test, *P* < 0.01). The abilities of fungal strains to transmit to *Arabidopsis thaliana* seeds and to develop necrosis on leaves were evaluated in this study or in indicated references, depending on the mutant; 0: unchanged comparing to the wild-type; –: decrease of the ability compared to the wild-type.*

### Modulation of Fungal Transcriptomes in Response to Water Stress

**FIGURE 1:**
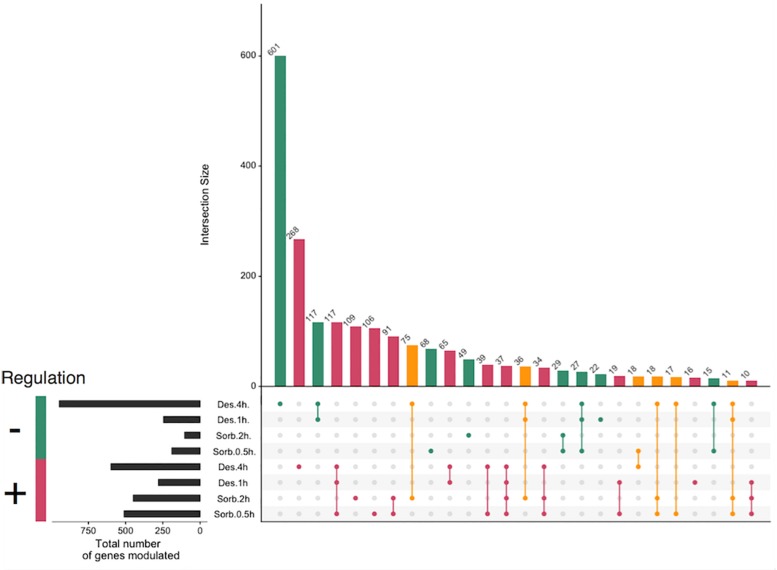
Global modulation of gene expression in *Alternaria brassicicola* in response to applied treatments. Sorbitol and desiccation treatments were compared to non-treated cultures at two time points 0.5 h, 2 h and 1 h, 4 h, respectively. Genes with a *P*-values ≤0.05 and a log2 ratio ≥0.7 or ≤–0.7 were considered as differentially expressed. Intersection with less than 10 genes were omitted. Colors displayed regulation compared to the control; red = up-regulated in all conditions, green = down-regulated in all conditions, orange = opposite regulation according to the considered condition.

In order to identify potential effectors of the response of *A. brassicicola* to dehydration stress, we first focused on the analysis of the transcriptional response in germinating conidia exposed to previously defined water stress (i.e., exposure to sorbitol 1.2 M for 0.5 and 2 h and exposure to silica gel beads for 1 h and 4 h). Each treated sample was compared to control sample and, thus, we produced four transcriptome data sets (each set was obtained from three biological replicates). We used *A. brassicicola* Nimblegen microarrays bearing one probe for each of the 10 633 ORFs predicted in the *A. brassicicola* automatically annotated genome database (JGI Genome Portal^1^). Only probes with a *P*-values ≤0.05 and a log2 ratio ≥0.7 or ≤−0.7 were considered as differentially expressed. The numbers of genes that were induced or repressed under each condition, together with a summary of those regulated in more than one condition, are shown in Figure 1. To validate our microarray results, the regulation of several genes by sorbitol or silica gel exposure was confirmed by quantitative PCR (Supplementary Figure S1).

Our data showed that, for all the treatments, with the exception of the 4 h-silica gel (4 h-desiccation) treatment, the number of up-regulated and down-regulated genes was less than 500 and 300, respectively. Most of these genes were found to be regulated in more than one condition, 37 were found to be induced in all 4 stress conditions while none were found to be repressed. The 4h-desiccation stress led to more profound transcriptomic modifications since 578 and 917 were induced or repressed compared to control condition, respectively. Moreover, this transcriptomic response seemed rather specific since more than 800 genes (268 up-regulated and 601 down-regulated genes) were specifically regulated after the fungus was exposed to this constraint. It should be noted that 157 genes, that were induced by the sorbitol exposure, were repressed following the silica gel treatment.

**FIGURE 2:**
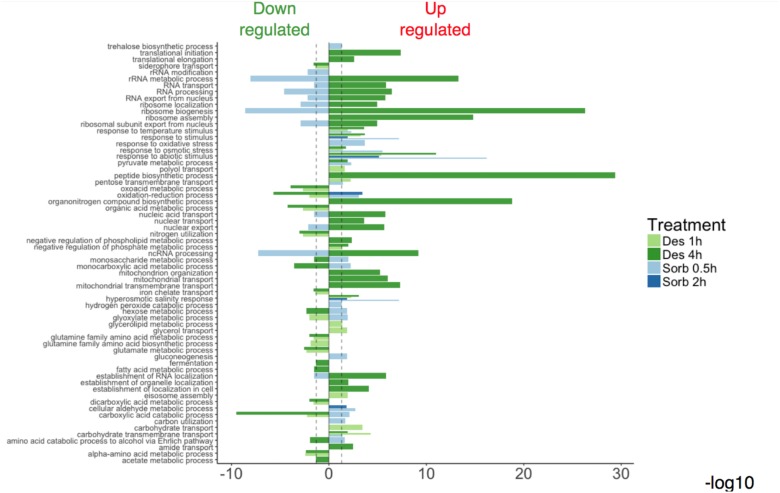
Relevant Gene Ontology Biological Process (GO-BP) enrichments obtained from modulated gene sets after exposure to water stresses.

The GO enrichment analyses showed that the enriched GO terms were not generally shared within the gene sets representative of each type of treatment (sorbitol or desiccation) (Figure 2). One exception was the GO categories corresponding to the response to oxidative and osmotic stresses, which were enriched in all the lists of induced genes. Another configuration was that enriched categories emerging from the 4h-desiccation up-regulated genes were also enriched categories emerging from the down-regulated gene lists after sorbitol exposure. These results seem to point out that the two types of treatment did not cause the same effects at the cellular level and induced quite different transcriptomic responses.

Enriched categories emerging from genes regulated following silica gel exposure were mainly related to the translation process, mitochondrial functions, and carboxylic acid and amino acids metabolic processes. Within the up-regulated genes, the main enriched GO terms identified in the sorbitol data sets were predominantly associated with a response to oxidative stress and to sugar metabolic process. Within the sorbitol repressed gene list, enriched GO terms referred to a wider range of cellular processes such as gene expression (Figure 2).

### Metabolomic Responses to Water Stress

**FIGURE 3:**
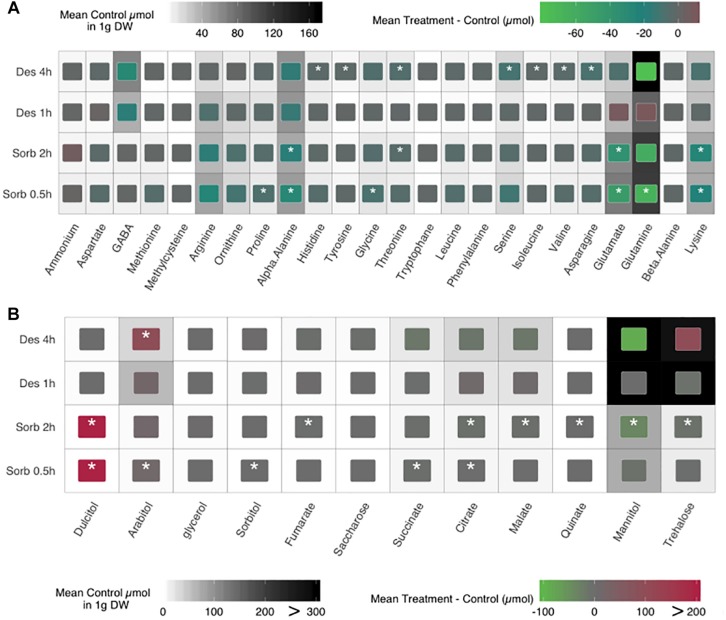
Impact of sorbitol and desiccation treatments on amino acid **(A)**, saccharides and sugar alcohols **(B)** contents of fungal cultures. The heat map background displays the mean control contents. Internal colored boxes display the mean difference between treatments and controls. Quantifications were performed from three separate samples. Asterisks indicate a significant difference between the mutant and the parental isolate (Welch test, ^∗^*p* ≤ 0.05).

As several enriched GO categories emerging from transcriptomic data were linked to amino acid metabolism and catabolism, we investigated whether the response to water stress in *A. brassicicola* resulted in an alteration of the cellular content in amino acids. Regardless of the hydric stress applied, the total amino acid concentrations were significantly lower for the treatments conditions compared to the control conditions (Welch test *P*-value = 5.955E^–08^, Supplementary Figure S2). Indeed, considering the 24 amino acids and derivatives that were quantified in this study, the mean ratio of amino acid concentration in the treated conditions compared to the respective controls decreased significantly for 17 and 12 amino acids (9 in common), regardless of time exposure for the sorbitol and the desiccation treatment, respectively (Figure 3A). Amino acid profiles that were differentially less accumulated compared to controls were quite similar after 0.5 and 2 h of sorbitol exposure with 9 shared amino acids (over 13). Glutamine, glutamate, alpha alanine, lysine and arginine concentration explained 70% [187.8/270.0 μmol.g^–1^ DW (dry weight)] and 78% (156.3/199.8 μmol.g^–1^ DW) of the amino acid variations for the sorbitol treatment at 0.5 and 2 h, respectively. For the 4 h desiccation condition, glutamine, GABA, alpha alanine and lysine explained 70% (134.7/194.6 μmol.g^–1^ DW) of the amino acid variations. No significant concentration differences were observed for the 1 h desiccation treatment.

Then, we focused on sugar content, since their intracellular accumulation is a well-known protective mechanism to cope with the water constraints (Dupont et al., 2014). Eight saccharides and sugar alcohols were quantified in treated and control conditions (Figure 3B). The total concentration of those compounds was found to be significantly different only in the case of comparison between sorbitol-treated and untreated conditions (Welch test *P*-value = 2.05E^–5^, Supplementary Figure S2) regardless of the time exposure. Unexpectedly, we observed a strong accumulation of some sugars (arabitol, trehalose, mannitol) in the control of the desiccation condition. This result could explain that no significant difference on the sugar cellular contents was observed between treated and untreated samples, except for an arabitol accumulation after a 4 h-treatment.

As expected, glycerol was accumulated in fungal cells exposed to sorbitol. Two other polyols, arabitol and dulcitol, were also strongly accumulated under these conditions. Conversely, the amounts of other polyols and the disaccharide trehalose were lower in cells treated with sorbitol than in untreated conditions.

### Hydrophilins Identification Through *in silico* Analysis

Hydrophilins could be defined as glycine-rich, highly hydrophilic disordered proteins. This criterion was used to search in *A. brassicicola* for putative hydrophilins among the whole protein set deduced from the recently published genome of *A. brassicicola* strain Abra43 (12,456 entries, Belmas et al., 2018). A total of 732 predicted proteins with an average hydrophilicity above one (GRAVY index lower than −1) were first selected and analyzed for their glycine content. The 210 predicted proteins with at least 8% glycine were retained and subjected to secondary structure prediction analysis. Almost three quarters of them (165) were predicted to adopt a coil configuration over more than 50% of their length. After removing mis-annotated and incomplete proteins and those that had an obvious match upon Blast analysis with proteins of known functions (except “stress response”), a final set of 107 candidate hydrophilins (c.a. 0.9% of the whole predicted proteome) was selected (Table 3). Interestingly, among proteins that had physicochemical features of hydrophilins but were discarded after Blast analysis, several displayed similarities with proteins involved in nucleic acids metabolic processes (Supplementary Table S1). Each of the 107 candidate hydrophilins was characterized by an index of significance, calculated based on the following formula: [- (gravy index −1) X (% G – 8) X (% rc – 50)] X 100, whose value varied from 3 to 36.512. Six putative homologs of fungal hydrophilins were found within the whole set of *A. brassicicola* hydrophilin-like proteins: HSP12 (AB01868/Abra01064 and AB10131/Abra12207), STF2 (AB03803/Abra04061) from *Sa. cerevisiae*, CON10 (AB02056/Abra00844 and AB01782/Abra01163) and CON6 (Abra02927) from *Neurospora crassa*, and the dehydrin-like proteins from *A. brassicicola* AbDhn1 (AB02513/Abra00274) and AbDhn3 (AB05365, AB05364/Abra08036). None of the other yeast hydrophilins had putative homolog in the *A. brassicicola* genome except NOP6 (AB08803/Abra04570) and WWM1 (AB05865/Abra11690) that were not selected due to a glycine content below 8% or a gravy index above −1, respectively. Similarly, due to an average hydrophilicity below the threshold value, the other *A. brassicicola* dehydrin-like protein AbDhn2 (AB08993/Abra04787) was not selected within the candidate hydrophilins set. This protein set also contains the putative homolog DNA-damage responsive DDR48 (AB03330/Abra05531) protein from yeast.

**TABLE 3:** Set of candidate hydrophilins identified from the *Alternaria brassicicola* genome sequence through *in silico* analysis.

**Prot ID**									
**Ab43 genome**	**ref genome**	**Gravy**	**Length**	**%G**	**%CC**	**Score**	**Blast**	**Stress induction**	**Proposed name**
Abra00077	AB02678	−1.11	100	11	51	34			
Abra00088	AB02665	−1.00	334	9.3	58	5		+	AbSih1
Abra00257	AB04427	−1.16	349	9.7	59	252			
Abra00274	AB02513	−1.07	408	9.1	72	172	DHN1	+	AbDhn1
Abra00844	AB02056	−1.07	114	15.8	69	1092	con10	+	AbSih2
Abra01053	AB01878	−1.02	77	15.6	62	213			
Abra01064	AB01868	−1.08	100	13	54	164	HSP12	+	AbSih3
Abra01163	AB01782	−1.17	75	17.3	54	625	con10		
Abra01374	AB01197	−1.06	124	12.9	79	791			
Abra01387	AB01185	−1.14	255	27.1	76	7186			
Abra01450	AB01144	−1.16	551	8.5	62	98			
Abra01572	AB01031	−1.10	327	10.1	60	207			
Abra01616	AB00988	−1.10	236	12.2	64	598			
Abra01703	AB00914	−1.28	849	12	72	2426			
Abra01742	AB00884	−1.07	443	9.7	54	47		+	AbSih4
Abra01976	AB00692	−1.24	486	9.5	63	474			
Abra02025	AB00652	−1.38	196	10.1	61	884			
Abra02123	AB00572	−1.04	142	9.9	59	65			
Abra02179	AB00532	−1.78	62	16.1	75	15873			
Abra02286	AB00441	−1.20	84	10.7	71	1114		+	AbSih5
Abra02383	AB00362	−1.04	1020	8.9	67	59			
Abra02582	AB00195	−1.16	120	10	66	523		+	AbSih6
Abra02745	AB10671	−1.63	323	14.6	65	6262		+	AbSih7
Abra02936	AB09442	−1.01	139	12.2	63	35			
Abra03033	AB09525	−1.02	270	11.1	61	56		+	AbSih8
Abra03243	AB04503	−1.27	122	13.9	53	474			
Abra03342	AB04427	−1.12	309	9.7	67	343			
Abra03378	AB04393	−1.44	200	19	77	13068			
Abra03462	AB04328	−1.36	340	18.5	60	3805			
Abra03538	AB04263	−1.01	358	9.8	64	28			
Abra03729	AB04091	−1.04	112	14.3	60	225			
Abra03785	AB04037	−1.21	417	10.8	63	781	PAL1		
Abra03984	AB03874	−1.33	494	10.1	71	1465			
Abra04061	AB03803	−1.13	155	9	62	153	STF2		
Abra04301	AB10372	−1.49	396	8.1	53	15			
Abra04434	AB08681	−1.04	578	19.7	63	676			
Abra04604	AB08829	−1.15	344	9	58	118			
Abra04882	AB09668	−1.20	146	8.2	52	8			
Abra05347	AB01286	−1.02	664	9.8	66	59			
Abra05531	AB03330	−1.96	314	13.7	90	21943	DDR48	+	AbSih9
Abra05532	AB03329	−1.24	397	27.2	69	8592		+	AbSih10
Abra05581	AB03281	−1.24	210	15.7	79	5253			
Abra05783	AB03114	−1.49	89	10.1	60	1026			
Abra05849	AB03052	−1.11	310	13.2	71	1180		+	AbSih11
Abra06033	AB02899	−1.06	267	9	56	34		+	AbSih12
Abra06151	AB02794	−1.14	138	16.7	68	2145		+	AbSih13
Abra06575	AB07491	−1.22	587	10.7	56	357		+	AbSih14
Abra06733	AB07371	−1.04	79	13.9	72	509			
Abra06831	AB10289	−1.08	87	24.1	87	4519			
Abra07098	AB04617	−1.15	49	8.2	69	56			
Abra07398	AB04822	−1.04	112	8.9	62	39			
Abra07543	AB04946	−1.41	59	8.5	64	285			
Abra07677	AB05060	−1.35	281	11.4	63	1535			
Abra07755	AB05132	−1.18	425	9.2	61	231			
Abra07777	AB05148	−1.08	132	10.6	70	437		+	AbSih15
Abra07844	AB05207/	−1.09	177	10.2	78	529			
Abra08036	AB05365 AB05364	−1.00	1314	10.3	68	7	DHN3	+	AbDhn3
Abra08116	AB05433	−1.24	383	18.5	62	3040		+	AbSih16
Abra08144	AB05453	−1.10	131	16	74	1964		+	AbSih17
Abra08312	AB01538	−1.25	178	12.4	70	2200			
Abra08733	AB06615	−1.15	265	9.1	63	216			
Abra09230	AB08588	−1.22	148	8.1	60	22			
Abra09944	AB07063	−1.55	93	8.6	53	99			
Abra09988	AB07108	−1.29	108	9.3	58	298			
Abra10303	AB09288	−1.17	233	9.4	54	97			
Abra10788	AB07911	−1.34	139	12.9	63	2186			
Abra10847	AB07968	−1.21	76	10.5	68	953			
Abra10981	AB08081	−1.13	206	8.7	51	9			
Abra11043	AB08134	−1.18	127	12.6	62	1008			
Abra11399	AB06102	−1.40	238	20.6	75	12640			
Abra11512	AB06021	−1.51	189	11.1	65	2379			
Abra11736	AB05830	−1.31	188	13.3	81	5016			
Abra11761	AB05809	−1.73	94	9.6	62	1393			
Abra12091	AB05542	−1.01	384	8.3	76	9			
Abra12172	AB05473	−1.88	224	12.1	81	11218			
Abra12188	AB10143	−1.18	326	10.4	80	1281			
Abra12207	AB10131	−1.03	101	14.9	58	186	HSP12	+	AbSih18
Abra12251	AB10094	−1.23	242	13.6	62	1522			
Abra09652		−1.27	62	11.3	72	1944			
Abra11726		−1.09	109	8.3	51	3			
Abra02927		−1.03	75	13.3	57	114	con 6		
Abra04037		−1.06	103	8.7	52	8			
Abra04374		−1.57	62	9.7	88	3709			
Abra04664		−1.14	180	14.4	62	1092			
Abra05405		−1.47	104	10.6	64	1722			
Abra06548		−1.45	164	10.4	59	971			
Abra12282		−1.46	159	12.6	60	2115			
Abra00223		−1.47	70	8.6	54	113			
Abra00730		−1.28	63	11.1	63	1126			
Abra01125		−1.01	44	9.1	79	44			
Abra02379		−1.01	73	20.5	53	41			
Abra02426		−1.67	52	13.5	57	2562			
Abra04371		−1.83	68	20.6	85	36512			
Abra04903		−1.44	69	8.7	53	93			
Abra05665		−1.05	60	8.3	65	22			
Abra06247		−1.06	51	9.8	62	131			
Abra07265		−1.56	52	9.6	71	1880			
Abra07528		−1.00	70	15.7	72	48			
Abra07587		−1.11	51	9.8	64	267			
Abra08211		−1.32	54	9.3	51	41			
Abra10089		−1.39	62	11.3	62	1552			
Abra10521		−1.52	52	9.6	71	1732			
Abra10564		−1.52	52	9.6	71	1732			
Abra10568		−1.40	58	8.6	72	526			
Abra11431		−1.10	52	9.6	53	47			
Abra12286		−2.04	66	10.6	69	5127			
Abra07568		−1.01	56	12.5	85	225			

### Expression of Hydrophilins During Osmotic and Hydric Stress

**FIGURE 4:**
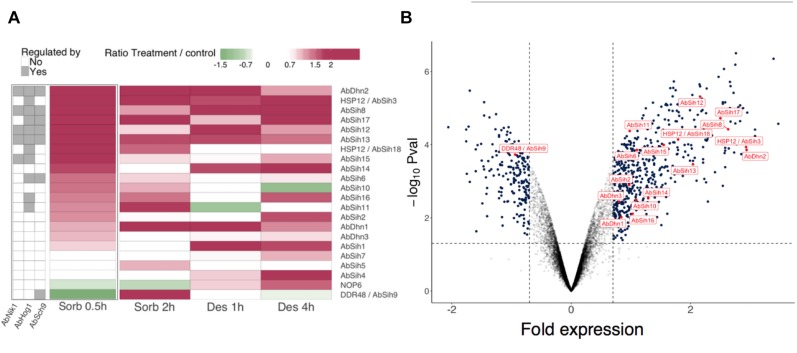
Gene expression of *Alternaria brassicicola* hydrophilins in response to sorbitol and desiccation treatments. Sorbitol and desiccation treatments were compared to non-treated cultures at two time points 0.5 h, 2 h and 1 h, 4 h, respectively. **(A)** For each gene, numerical data were transformed into color-grid representations in which the fold gene expression induction (log2 values) is represented by a colored scale. In addition, hydrophilin gene expression were evaluated in mutant strains Δ*abnik1*, Δ*abhog1* and Δ*absch9* exposed to 1.2 M sorbitol for 0.5 h. The left part of the panel **(A)** highlights the possible regulation of hydrophilin gene expression by one or several of these protein kinases. **(B)** Volcano plot showing fold inductions of genes in the wild-type strain exposed to 1.2 M sorbitol for 0.5 h. The hydrophilin encoding genes are labeled and identified with red dots. The threshold values (*P*-values ≤0.05 and a log2 ratio ≥0.7 or ≤–0.7) are indicated by dash lines.

Then, we explored the expression pattern of genes encoding putative hydrophilins from transcriptome data sets generated after exposing germinating conidia of *A. brassicicola* to 1.2 M sorbitol and to low relative humidity (silica gel beads). These data sets were obtained using microarrays designed based on the genome of the *A. brassicicola* ATCC 96836 strain available on the JGI Genome Portal (see footnote 1). This genomic sequence contained 10,514 predicted genes and the proportion of gaps between scaffolding boards in this sequence was relatively high (estimated to 5.2% by Dang et al., 2015). As described above, the *in silico* analysis was performed from the recently published genome of *A. brassicicola* strain Abra43 (Belmas et al., 2018^2^^,^^3^), which is a higher quality sequence containing 12,456 predicted protein-coding genes. Considering these proteome differences, expression patterns were available only for 78 putative hydrophilins that matched with a predicted ORF from the ATCC 96836 strain proteome. Under these conditions, 17 of the 78 (21.8%) candidate hydrophilin genes were up-regulated by the sorbitol treatment (Figure 4A). For all of them, except AB03330 and AB00441, an induced expression was observed at 0.5 h (Figure 4B). Moreover, 13 candidate hydrophilin genes were up-regulated by the desiccation treatment, 10 of them being also up-regulated upon the sorbitol treatment. All of these proteins were named with the abbreviation “SIH” for “stress-induced hydrophilin-like” (Table 3).

To gain insight on the possible regulation of hydrophilin genes expression by different kinases known to regulate the osmostress response, the expression profiles of hydrophilin genes in the Δ*abhog1*, Δ*abnik1* and Δ*absch9* mutants were compared with those in the wild-type parental strain under both control condition and after exposure to sorbitol for 0.5 h (Figure 4). This analysis revealed that, among the sorbitol-inducible hydrophilin genes, 6 were dependent of at least two of the three kinases for their expression. Two were regulated by AbHog1 and AbNik1, two others by AbHog1- and AbSch9 and three others by AbHog1, AbNik1 and AbSch9. The gene encoding AbDhn2 was also induced by the treatment and was found Hog1-Nilk1-Sch9-dependent.

### Role of Selected Hydrophilins in *A. brassicicola*

Two putative hydrophilins (AbSih3/AB01868 and Absih15/AB05148) were selected for further studies aiming to investigate their function in *A. brassicicola*. Knockout mutants deficient for AbSih3 and Absih15 (called Δ*absih3* and Δ*absih15*), respectively, were constructed by replacing the respective ORFs with a hygromycin B resistance cassette (Supplementary Figure S3). For each targeted gene, two replacement mutants were selected using a PCR screen and further purified by two rounds of single-spore isolation. In all further experiments, the phenotypic characters for transformants of the same genotype were not found to be significantly different and the phenotyping values that we displayed were calculated by taking into account both transformants.

**FIGURE 5:**
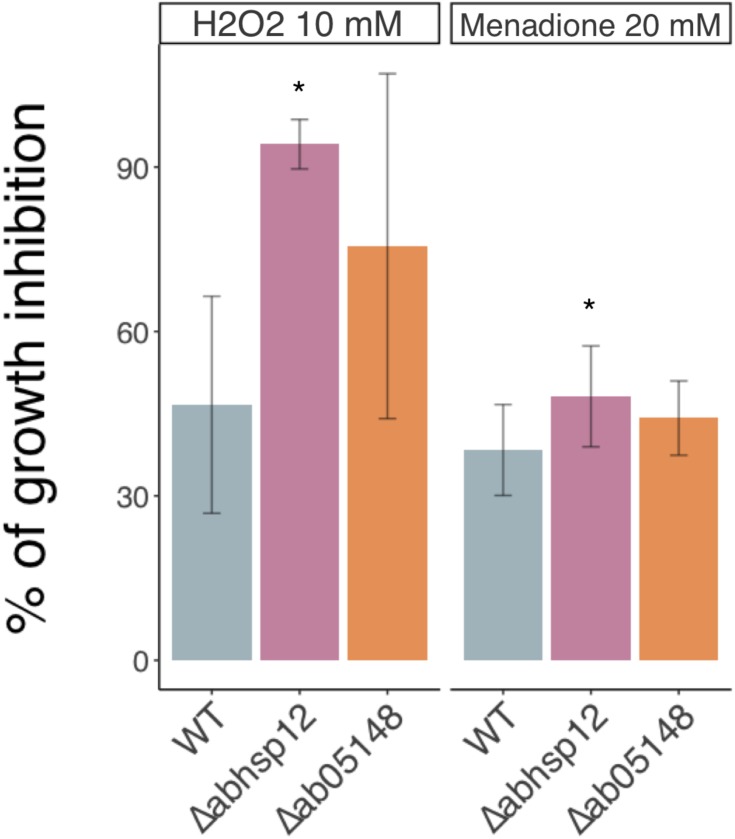
Growth inhibition rates of the wild-type strain (WT) and hydrophilin deficient mutants in the presence of H_2_O_2_ (10 mM) and menadione (20 mM). The results are expressed as the percentage of inhibition in treated samples compared to the control without additive. Conidia were used to inoculate microplate wells containing standard PDB medium that was supplemented with the appropriate test substance. Growth was automatically recorded for 30 h at 25°C using a nephelometric reader (see section “Materials and Methods”). Each genotype was analyzed in triplicate and the experiments were repeated at least three times per growth condition. Error bars indicate standard deviations. Asterisks indicate a significant difference between the mutant and the parental isolate (Dunn control test, ^∗^*p* ≤ 0.01).

Although the expression of these genes was induced in germinating conidia exposed to previously defined water stress (i.e., exposure to 1.2 M sorbitol and to silica gel beads), the knock-out approach showed that only the *AbSih3* deletion had an impact on phenotype, mainly on oxidative stress tolerance. The corresponding deficient single mutants were indeed characterized by a slightly increased susceptibility toward oxidative stress generated by exposure to menadione and H_2_O_2_ compared to the wild-type (Figure 5). No significant difference in susceptibility to other tested stresses (exposure to PEG −0.7 MPa, 350 mM NaCl, 1.2 M sorbitol) was found compared to the wild-type (data not shown).

**FIGURE 6:**
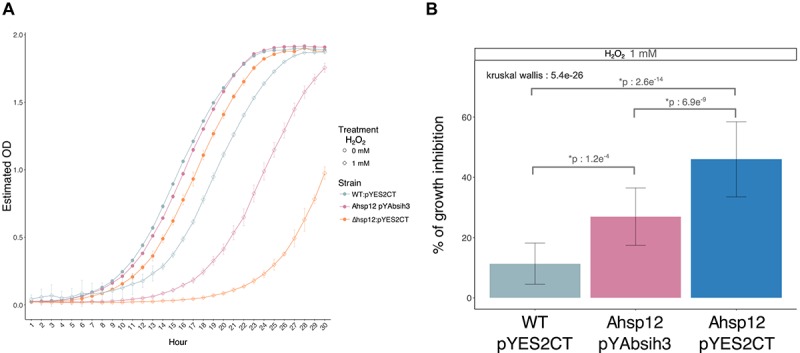
Complementation of yeast Δ*hsp12* mutants with *AbSih3* coding sequences. **(A)** Growth curves of *Saccharomyces cerevisiae* (parental WT strain and Δ*hsp12* mutants) cells transformed with empty vector (pYES2CT) or recombinant vectors (pYAbsih3) exposed to 1 mM H_2_O_2_ or to water. **(B)** Comparisons of growth inhibition rates obtained for each yeast strain (Wilcoxon test, ^∗^*p* < 0.001).

Consistently, a similar higher susceptibility to H_2_O_2_ of the *Sa. cerevisiae hsp12* null mutant was observed (Figure 6). To confirm that *AbSih3* correspond to true functional homolog of the yeast *hsp12* gene, complementation of *hsp12* mutant null mutant was carried out with relevant recombinant pYES plasmids. As shown in Figure 6, *AbSih3* successfully complemented the *Sa. cerevisiae hsp12* phenotypes, even if the growth of Δ*hsp12* cells expressing AbSih3 was not restored to a level comparable to that of the WT strain containing the empty vector pYES2 on media supplemented with 1 mM H_2_O_2_.

**FIGURE 7:**
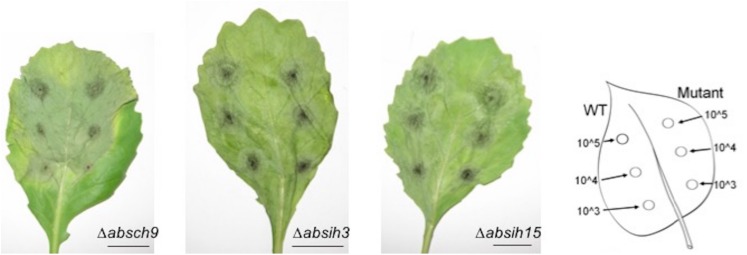
Representative symptoms at 5 dpi obtained by inoculation of wildtype, Δ*absch9*, Δ*absih3*, and Δ*absih15* on cabbage leaves. Leaves were inoculated with 5 μL drops of conidia suspension (10^5^, 10^4^ or 10^3^ conidia/mL in water). As shown on the right side, mutants were inoculated on the right part of the central vein and compared on the same leaf with the parental strain (inoculated on the left part of the central vein). Bars = 2 cm.

Then, the virulence of the *A. brassicicola* wild-type, Δ*absih3* and Δ*absih15* strains were compared on *B. oleracea* and *A. thaliana* host plants during host vegetative tissue infection or during the seed transmission process. *Brassica oleracea* leaves were inoculated with drops of conidia suspension (10^5^, 10^4^, or 10^3^ conidia/mL). As shown in Figure 7, the wild-type and KO strains were all able to produce typical symptoms and their aggressiveness was not impaired. Regardless of the inoculated strain, first necrotic symptoms appeared on leaves at 3 days post-inoculation (dpi). Necrotic areas enlarged and surrounded themselves with chlorotic halos at 6 dpi. During late stages of infection, the fungus produced conidia on the surface of necrosis.

**FIGURE 8:**
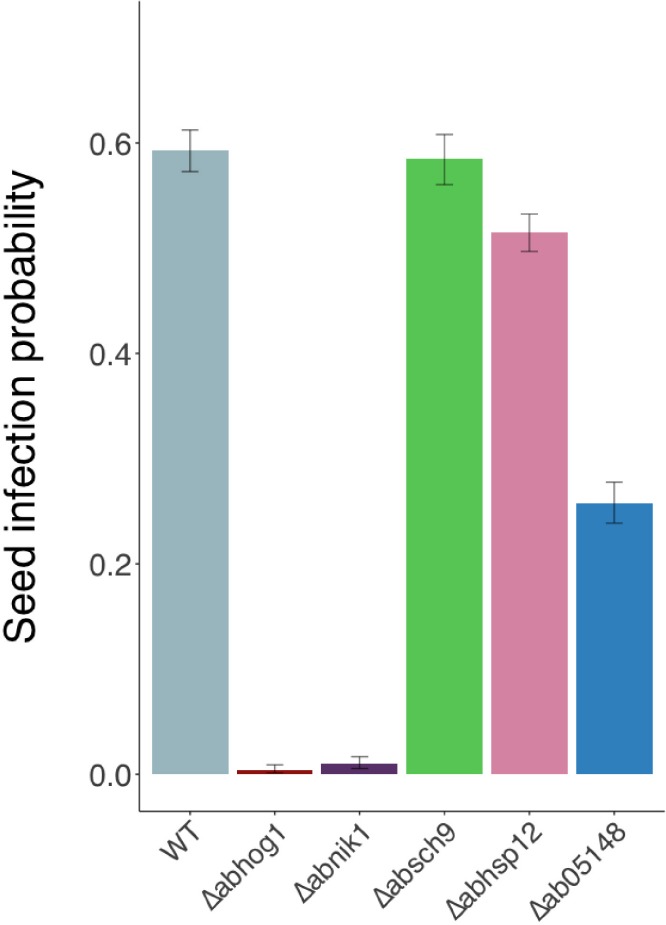
Transmission capacity of *Alternaria brassicicola* wild-type (WT) and null strains to *Arabidopsis thaliana* seeds (Ler ecotype). The seed transmission capacity were measured as described by Pochon et al. (2012). The five youngest siliques of at least five plants were inoculated with each fungal genotype and the experiment was repeated twice. Contaminated siliques were harvested 10 dpi. After dissection, seeds were incubated separately on PDA medium for 2 days. A seed was considered contaminated when incubation resulted in typical *Alternaria brassicicola* colony development. For each inoculated fungal genotype, the seed infection probability was evaluated from at least 1000 seeds. Values represent infection probabilities with 95% confidence interval.

Using the model pathosystem previously described by Pochon et al. (2012) for investigating seed transmission in *Arabidopsis* plants, the ability of the hydrophilin mutants to transmit to seeds was compared with that of the wild-type. As a positive control, we also analyzed the behavior of the osmosensitive fungal mutants Δ*abnik1* and Δ*abhog1*, which were previously described as being strongly altered in their seed transmission capacity (Pochon et al., 2012). Silique inoculation with the wild-type and hydrophilin mutant strains resulted in the development of mycelium and typical lesions covered with conidia on siliques within a few days after inoculation. Seeds were then individually harvested and plated on PDA medium for a microbiological examination. As expected, a strong decrease in seed transmission rate was observed for Δ*abnik1* and Δ*abhog1* compared to the wild-type parental strain (Figure 8). Interestingly, the seed transmission capacity of the Δ*absch9* mutant was not altered although this strain was found to be sensitive to sorbitol and desiccation. While the deletion of *AbSih3* had a slight significant impact on seed transmission, a stronger decrease was observed for the Δ*absih15* mutant, for which the seed transmission rate was reduced by a factor two compared to the wild-type strain.

## Discussion

The ability of *A. brassicicola* to cope with water-stress constitutes a key factor for successful seed colonization and thus accomplishment of its entire infectious cycle (Iacomi-Vasilescu et al., 2008; Pochon et al., 2012, 2013). Highly hydrophilic and disordered glycin-rich proteins containing repeated DPR-motifs called dehydrins were identified in *A. brassicicola* and might correspond to effectors of the response to water stress (Pochon et al., 2013). Based on their physicochemical features, dehydrins are considered as members of a larger protein family called hydrophilins initially described in *Sa. cerevisiae* (Garay-Arroyo et al., 2000). In an attempt to identify additional effectors of the response of *A. brassicicola* to water stress, the proteome deduced from the recently published genome of *A. brassicicola* strain Abra43 (Belmas et al., 2018) was first screened for proteins with characteristic features of hydrophilins. Candidate hydrophilins accounted for 0.9% of the whole predicted proteome. In comparison, hydrophilins represented only 0.2% of the yeast proteome (Garay-Arroyo et al., 2000) although the selection criteria were less stringent, i.e., proteins with lower glycine content were included. This difference might reflect a family expansion associated with environmental adaptation and/or fungal pathogenesis as it was proposed for other sets of proteins, e.g., secreted proteins, enzymes for secondary metabolites production (Soanes et al., 2008; Ohm et al., 2012). However, this assumption remains to be demonstrated and would require a more extensive search for hydrophilin repertoires in fungi with various lifestyles.

The fact that two of the three previously described dehydrins and additional homologs of fungal hydrophilins were included in the repertoire of candidate hydrophilins validated our screening process. Garay-Arroyo et al. (2000) proposed that hydrophilins that were not induced by water-stress conditions should not be considered as true hydrophilins. Thus, we analyzed changes in transcript levels in *A. brassicicola* germlings exposed to two kinds of water-stress: hyperosmosis (exposure to 1.2 M sorbitol) and desiccation (exposure to silica gel beads). Using gene expression array, which was designed from a previous genome sequence with a lower quality, we defined the expression patterns of 78 genes (over 107) encoding these putative hydrophilins. Twenty candidate hydrophilin genes were up-regulated either by the sorbitol treatment or by the desiccation stress, and, among them, ten were induced by both treatments. So, according to the hydrophilin definition given by Garay-Arroyo et al. (2000), we identified at least 20 hydrophilins in *A. brassicicola*. However, we can reasonably assume that their number is higher since we did not have access to the gene expression of 29 other genes encoding putative hydrophilins.

In this study, strains deficient for AbSih3 exhibited a slightly lower susceptibility to oxidative stress compared to the wild-type strain. No significant difference in susceptibility to other tested stresses (exposure to PEG, NaCl and sorbitol) was found for Δ*absih3* and Δ*absih15*. Given the large number of hydrophilins that were co-expressed in this organism, functional redundancy may occur between some of these proteins. This process would explain the scarcity of significant phenotypes that we obtained from single deficient mutants since the presence of other hydrophilin genes would compensates for the loss. For instance, AbSih3 displays high sequence similarity to AbSih18 (AB10131), and both may be considered as homologs of the *Sa. cerevisiae* HSP12 hydrophilin, even if AbSih3 only partially compensated the HSP12 loss in yeast. The existence of such a functional redundancy was also previously observed between two dehydrins AbDhn1 and AbDhn2 in *A. brassicicola*. The double mutant ΔΔ*abdhn1-abdhn2* was severely compromised in its pathogenicity while the respective single mutants were not affected (Pochon et al., 2013).

Despite this probable functional compensation, we showed here that the hydrophilins Absih3 and Absih15 could be considered as pathogenic factors since the corresponding null strains were affected in its seed transmission ability (more strongly for the Δ*absih15* strain). This result confirmed our initial assumption that an efficient fungal adaptive response to severe hydric stress conditions or to oxidative stress strongly influenced the seed transmission ability. This is consistent with the fact that, during seed colonization, fungi are exposed to a gradual decrease in the water potential occurring in maturing reproductive organs and that they have to cope with this particular stress to be efficiently transmitted to seeds and complete their infection cycle. In the same vein, we showed in this study or in previous works that the class III HK AbNik1 and the MAP kinase AbHog1, two components of the high osmolarity pathway, were required for an effective seed colonization by *A. brassicicola* (Pochon et al., 2012). The Δ*absch9* mutant was also found to be sensitive to sorbitol and desiccation but, unexpectedly, its seed transmission capacity was not altered. The fact that this mutant strain did not exhibit a high susceptibility to oxidants suggested that the fungal ability to overcome oxidative stress would be an additional or the main key mechanism for an efficient seed infection process. This hypothesis is consistent with the fact that oxidative damages produced by free radical species are considered to be one of the major causes of desiccation injuries (Dupont et al., 2014).

A final point raised from these results is that several *A. brassicicola* mutants (Δ*abnik1, Δabsih3* and Δ*absih15)* were affected in their transmission to *A. thaliana* seeds, although their aggressiveness on host vegetative tissues remained intact. This fact confirms the specificity of some molecular mechanisms controlling transgenerational transmission of fungal pathogens (Pochon et al., 2012) and validates the strategy to gain further insight into these mechanisms for the development of specific seed disease control strategies.

In addition to the induction of genes encoding hydrophilins, we identified other metabolic clues related to the activation of an adaptive response to water stress in *A. brassicicola*. First, the cellular content in amino acids was strongly disturbed following the application of both stresses. The amino acid levels were indeed significantly reduced in treated cells compared to controls. In line with this result, several enriched GO categories emerging from transcriptomic data were linked to amino acid metabolism and catabolism. Moreover, amino acid synthesis was also found to be strongly down-regulated in response to the osmotic stress treatment in another *Dothideomycete* fungus, the wheat pathogen *Stagonospora nodorum* (Lowe et al., 2008). As reported in plants (Hildebrandt et al., 2015), the degradation of amino acids may lead to the production of an additional energetic resource, that could be useful to cope with adverse conditions, by providing intermediates of the tricarboxylic acid cycle and finally contributing to ATP production. Moreover, the oxidation of some amino acids feeds electrons into the mitochondrial electron transport chain. Most reaction steps of the catabolic pathways occur either in the mitochondria or cytosol. Consistent with this hypothesis, several enriched GO terms referred to mitochondria organization, carboxylic acid metabolism and to oxidation-reduction process. Regarding the response to desiccation, as a GO enrichment of functions related to the genesis and transport of ribosomes and, more generally, to translation was observed, we could assume that a strong induction of the translation process would trigger a depletion of the cellular amino acid pool. So, the mechanisms leading to the decrease of the amino acid could depend on the type of applied stress.

An intriguing result from our data was that no significant difference in the amino acid concentrations was observed for the 1 h-desiccation treatment compared to the respective control. A more precise inspection showed that, for this condition, both control and treated samples showed a similar low amino acid content (Supplementary Figure S2). In this assay, germlings grown on cellophane were taken off from the PDA plate and directly transferred to sealed boxes (without additional agar medium) with or without silica gel beads. In the control box (without silica gel), we suspect that germlings were also exposed to a strong constraint in the early time of exposure, as a period of 4 h was required to gradually reach a RH value of about 90%. Therefore, although this experimental device triggered a specific regulation at the transcriptome level (Figures 1, 2), this does not appear to be sufficient to reveal significant differences in amino acid accumulation between control and treated samples at this analysis time.

In the sorbitol-exposed samples, a strong increase in global sugar contents was observed compared to sorbitol control. Interestingly, the accumulation of non-reducing disaccharides, such as trehalose, did not seem to be favored in *A. brassicicola* germlings under the sorbitol stress conditions. On the contrary, the contents of three polyols, glycerol, arabitol and dulcitol, were mainly impacted by this stress, suggesting that they function as intracellular osmolytes to counteract the effects of decreases in the cell volume and the loss of turgor pressure (Sánchez-Fresneda et al., 2013; Dupont et al., 2014). This result was consistent with the increased sensitivity of the Δ*abhog1* mutant to osmotic stress since the MAP kinase Hog1 is known to be required for endogenous glycerol production in several fungal species (Brewster and Gustin, 2014; Hohmann, 2015). However, its involvement in the synthesis of other protective compounds, such as arabitol or dulcitol, is still unclear (Kayingo and Wong, 2005; Sánchez-Fresneda et al., 2013). Arabitol has been described as part of a set of polyols which is developed to counteract environmental challenges in several yeasts and filamentous fungi (Pascual et al., 2003; Sánchez-Fresneda et al., 2013). For instance, arabitol, and to a lesser extent glycerol, were found to accumulate in response to the osmotic stress treatment in the wheat pathogen *St. nodorum* (Lowe et al., 2008) or in the rice pathogen *Magnaporthe grisea* (Dixon et al., 1999). Conversely, the role of dulcitol in stress responses remains highly confidential and has been reported on rare occasions, for instance in the marine fungus *Cirrenalia pygmea* (Ravishankar et al., 2006).

In addition to its involvement in the synthesis of compatible solutes, we showed here that the HOG pathway, mainly regulated by the MAP kinase AbHog1 and the upstream HK AbNik1, also acted as an expression regulator of some hydrophilin encoding genes. The expression profiles of hydrophilin genes in the Δ*abhog1*, Δ*abnik1*, and Δ*absch9* mutants were indeed compared with those in the wild-type parental strain after exposure to sorbitol for 0.5 h. Of the 17 proteins that were induced under these conditions, 11 and 5 were found to be under the control of AbHog1 and AbNik1, respectively. This result, associated with the weakness of Δ*abnik1* and Δ*abhog1* to survive on reproductive organs, highlighted that the HOG pathway is a major target for controlling both the synthesis of osmolytes and hydrophilins, and thus limiting the transmission of fungal pathogens to seeds. Unexpectedly, AbDhn1 and AbDhn3 were not part of this set of HOG-regulated proteins although Pochon et al. (2013) previously reported that these two proteins (in addition to AbDhn2) were targets for the AbHog1 signaling cascade when the fungus was exposed to a saline (NaCl) stress. This suggests a possible differential regulation of hydrophilins depending on the applied constraint.

Following a 0.5 h-sorbitol exposure, six other hydrophilins were also found to be AbSch9-dependent. As a major downstream effector of the target of rapamycin (TOR) complex 1, the Sch9 protein kinase was proposed to play multiple roles in stress resistance and nutrient sensing in *Sa. cerevisiae* (Pascual-Ahuir and Proft, 2007; Huber et al., 2009). However, the functions of *Sch9* orthologs in filamentous fungi have been poorly characterized to date. In *Fusarium graminearum*, Gu et al. (2015) showed that FgSch9 served as a mediator of the TOR and HOG pathways and played important roles in regulating vegetative differentiation, secondary metabolism and stress responses. We showed here that both hydrophilins AbSih3 and AbSih15 were necessary factors for an optimal *A. brassicicola* transmission to seeds but their expression was not found to be regulated by AbSch9 (regulation by AbHog1 and AbHog1-AbNik1, respectively). This result, associated with the fact that the seed transmission capacity of the Δ*absch9* mutant was not affected, suggested that this kinase (unlike AbHog1 and AbNik1), although involved in resistance to sorbitol and desiccation, is not a major player in transgenerational transmission of fungal pathogens.

## Materials and Methods

### Strains and Culture Conditions

The *A. brassicicola* wild-type strain *Abra43* used in this study was previously described (Sellam et al., 2007). For routine culture, *A. brassicicola* was grown and maintained on potato dextrose agar (PDA). The genome of *Abra43* strain was recently published and is available following this link https://bbric-pipelines.toulouse.inra.fr/myGenomeBrowser? (Carrere and Gouzy, 2017).

For transcriptomic analyses, exposure to sorbitol (osmotic stress) or to low relative humidity (desiccation stress) were achieved as follows. Conidia were first incubated for 24 h on cellophane disks overlaid on solid PDA medium. Germlings and the underlying cellophanes were then taken off from the agar plate and transferred either to a second PDA medium supplemented or not with 1.2 M sorbitol for 0.5 or 2 h.

The desiccation stress was achieved by applying a drastic decrease in relative humidity (RH) to the germlings (obtained as described below) by exposure to silica gel beads in sealed boxes for 0.5 or 4 h. Immediately after deposition of germlings (and the underlying cellophanes) in boxes, a strong RH increase was observed. In the control box (without silica gel), a gradual increase up to a RH value of about 90% in 4 h was observed. With silica gel beads, the opening of the boxes resulted in a rapid RH increase up to 20% followed by a decrease to a RH value of 1% in less than 0.5 h.

To evaluate the level of stress for each treatment applied to fungal cells and considering that the reference strain is naturally tolerant to water stress (Iacomi-Vasilescu et al., 2008), we additionally analyzed the effect of treatments on three mutant strains (derived from the wild-type Abra43 strain) for which we hypothesized a higher sensitivity to water stress. Two mutant strains (Δ*abnik1* and Δ*abhog1*) were described in previous studies (Dongo et al., 2009; Joubert et al., 2011) and were deficient for genes encoding key regulators of the HOG pathway, the HK AbNik1 and the MAP kinase AbHog1, respectively. The third mutant was obtained in this study and was deficient for the kinase AbSch9, which is also known to be involved in the fungal response to osmotic constraints (Pascual-Ahuir and Proft, 2007). Under non-stress conditions, no significant differences in mycelium growth were detected in any of the tested mutants as compared to the wild-type (data not shown). To assess the impact of low RH exposure on *A. brassicicola*, viability tests were conducted on young hyphae of wild-type and mutant strains. About 200 conidia were first incubated for 24 h on cellophane disks overlaid on solid PDA medium. Then, germinated conidia were transferred for 4 days to sealed boxes containing or not silica gel beads. The cellophane membranes were then removed from the boxes and deposited again on PDA medium. The survival rate was calculated by counting the number of mycelial colonies (derived from treated or untreated germlings) after 3 days of incubation at 24°C. As previouslys reported (de Lima Alves et al., 2015; Wang et al., 2017), exposure to these two treatments induces water stress in fungal cells. Exposure of wild-type and mutant strains to 1.2 M sorbitol were performed following the procedure described below. The radial growth was calculated after 6 days of incubation.

To study hyphal growth in liquid media, conidial suspensions (10^5^ conidia/mL, final concentration) were inoculated onto microplate wells containing the considered substance (10 mM H_2_O_2_ or 20 mM menadione) at the desired concentrations in potato dextrose broth (PDB, Difco) in a total volume of 300 μl. Fungal growth was monitored automatically over a 30 h period with a laser-based microplate nephelometer (NEPHELOstar, BMG Labtech) as described by Joubert et al. (2010). For each condition, the area under the growth curve representative of the lag phase was used to calculate the percentages of growth inhibition under stress conditions compared with standard growth conditions (PDB medium without additive), as described by Gaucher et al. (2013). At least three replicates were conducted per treatment.

### RNA Extraction and Microarray Analysis

Total RNA was extracted using the nucleospin RNAplant kit (Macherey Nagel, Düren, Germany) and 400 ng were amplified using the Ambion messageAmp II (Ambion, Austin, TX, United States) according to the manufacturer’s instructions. Five micrograms of amplified RNA were retro-transcribed with 400 U of Superscript II reverse-transcriptase (Invitrogen Corp., Carlsbad, CA, United States) and labeled with 1.5 mmol of Cyanine-3 (Cy3) or Cyanine-5 (Cy5) (Interchim, France) and then purified using NucleoSpin Gel and PCR Clean-up column kits (Macherey-Nagel, GmbH and Co., KG, Germany). Purified and labeled cDNA were quantified using a NanoDrop ND-1000. Corresponding Cy3- and Cy5 labeled samples (30 pmol) were combined and cohybridized to the Abra _v1.0 12 × 135K arrays on a NimbleGen Hybridization System 4 (mix mode B) at 42°C overnight. The Abra_v1.0 chip was *in situ* synthesized by Nimblegen (Madison, WI) and contained 10,633 60-mer oligoprobes that were designed from the *A. brassicicola* automatically annotated genome database^4^, containing one probe per sequence. Slides were washed, dried, and scanned at 2 μm resolution and high sensitivity with a Roche-NimbleGen MS200. DEVA Software version 1.2.1 was used to extract pair data files from the scanned images. Three biological replicates were analyzed per comparison using the dye-switch method. Statistical analyses of the gene expression data were performed using the R language, version 2.14, and the Linear Models for Microarray Analysis package (Smyth et al., 2005) from the Bioconductor project. For the preprocessing step, data were normalized by the LOWESS method. Log ratio and log intensity were calculated before differential expression analyses and performed using the lmFit function and the Bayes-moderated Student’s *t*-test in a linear model for microarray analysis. Probes with *P*-value <0.05 were considered as differentially expressed. Gene expression datasets were deposited in the Gene Expression Omnibus (GEO)^5^.

### Quantitative PCR

Total RNA was extracted as described above. Amplification experiments were conducted as previously described (Joubert et al., 2011). The relative quantification analysis was performed using the ΔΔCt method (Winer et al., 1999). All the primer sequences used in real-time quantitative PCR are presented in the Supplementary Table S2.

### Generation of Targeted Gene Knockout Mutants and Fusion Strains

We followed the procedure described by Pigné et al. (2017). Gene replacement cassettes were generated with the *Hph* gene cassette from pCB1636 (Sweigard et al., 1995) conferring resistance to hygromycin B. The sets of primers used to amplify the 5′ and 3′ flanking regions of each targeted gene are presented in the Supplementary Table S2. The replacement cassettes were used to transform *A. brassicicola* protoplasts as described by Cho et al. (2006). Primer combinations used to confirm integration of the replacement cassette at the targeted locus were presented in Supplementary Figure S3.

### Yeast Complementation Assays

Complementation assays were performed in *Sa. cerevisiae* cells (strains: parental BY4743 Acc#Y20000, ΔHSP12 Acc#Y27070, Euroscarf, Germany) as described by Joubert et al. (2011). Sequences encoding AbSih3 were amplified by PCR with primer pairs AbSih3-Bam (5′- **GGATCC**AAAAATGACCGACGCATTCCGC-3′) – AbSih3-Eco (5′- **GAATTC**CCATTCCAAGGGCGTTTTTGG-3′). Unique BamH1-*Eco*RI restriction sites (indicated in boldface) were thus introduced at the upstream start and downstream of the coding sequences, respectively and were used to clone the PCR product into pGEM-T (Promega) and then into the yeast expression vector pYES2CT (Invitrogen) to generate the recombinant plasmid pYAbhsp12. Sensitivity to 1 mM H_2_O_2_ was analyzed based on microcultivation in 300 μl liquid medium. Growth in microplates was automatically recorded using a spectrophotometer (Spectrostar Nano, BMG Labtech) at 30°C.

### Infection Assays

Leaf infection assays were performed on *B. oleracea* plants (var. Bartolo) as described by Joubert et al. (2011). The experiment was repeated twice and involved at least 10 leaves inoculated by each fungal genotype.

Seed infection assays and seed contamination assessments were performed as described by Pochon et al. (2012). At least five plants per fungal genotype were inoculated and the experiment was repeated twice.

### Metabolic Extraction and Amino Acids and Sugar Analysis

Metabolic extractions were made on 15 mg of dry powdered samples. Procedures for methanol–chloroform–water-based extractions as well as determination of amino acid and carbohydrate contents were described by Thouvenot et al. (2015).

## Author Contributions

GN’G carried out the sample preparation and microarray analyses. RR, BI, and MM involved in the sample preparation for metabolic analysis and performed the quantitative PCR. NB-S carried out the yeast complementation assays. CM, MM, and CA-B involved in the construction and phenotyping of *Alternaria brassicicola* mutant strains. PS and TG conceived the study and participated in the design of the experiments as well as in the analysis of the results. RL and BP involved in sugar analyses from fungal samples. SP and J-PR involved in the gene expression analyses. CC, MM, FB, BH, AK, and BI involved in the pathological tests.

## Conflict of Interest Statement

The authors declare that the research was conducted in the absence of any commercial or financial relationships that could be construed as a potential conflict of interest.

## Footnotes

1 https://genome.jgi.doe.gov/Altbr1/Altbr1.home.html

2 https://www.ncbi.nlm.nih.gov/nuccore/PHFN00000000

3 https://bbric-pipelines.toulouse.inra.fr/myGenomeBrowser?public=1

4 https://genome.jgi.doe.gov/Altbr1/Altbr1.home.html

5 https://www.ncbi.nlm.nih.gov/geo/query/acc.cgi?acc=GSE133507

## Supplementary Material

FIGURE S1 Comparison of transcript levels obtained by microarray or RT-qPCR methods. The expression of 9 genes was monitored in response to sorbitol and desiccation stresses. The fold inductions (log2 values) are presented in **(A)** and the correlation plot in **(B)**.

FIGURE S2 Total organic contents in a 1 mg sample of dried fungal cultures sample exposed or not to sorbitol and desiccation treatments. **(A)** Total contents of amino acids. **(B)** Total content of saccharides and sugar alcohols. Colors display the treatments. Box plots show the median, 1st and 3rd quantiles (*n* = 3). ^∗^denotes a significant difference (*P*-value <0.001, Welsh test).

FIGURE S3 Generation of null mutants by homologous recombination. **(A)** Schematic representation of the deleted locus in the wild-type and the replacement construct with the Hyg B resistance cassette (*Hph* gene). Arrows indicate the position of primers used for the cassette generation and for PCR screening of mutants. **(B)** Gel electrophoresis of PCR products obtained from template DNA of the wild-type, Δ*absch9*, Δ*absih3* and Δ*absih15* strains with the indicated primer pairs. Molecular sizes (kb) were estimated based on a 1 kb ladder (lane M, NEW ENGLAND BioLabs^®^ Inc.). A supplementary PCR was done with the primer pair ITS1/ITS4 as a positive control to assess the quality of the wild-type template DNA.

TABLE S1 List of predicted sequences from the *Alternaria brassicicola* genome exhibiting physicochemical features of hydrophilins and an obvious match upon Blast analysis with proteins of known functions.

TABLE S2 List of primers used in this study.
