# Supplementary figures and images for: Responses to Hydric Stress in the Seed-Borne Necrotrophic Fungus Alternaria brassicicola
Source: Front Microbiol. 2019 Aug 30;10:1969. doi: 10.3389/fmicb.2019.01969 (PMC6730492; doi:10.3389/fmicb.2019.01969)

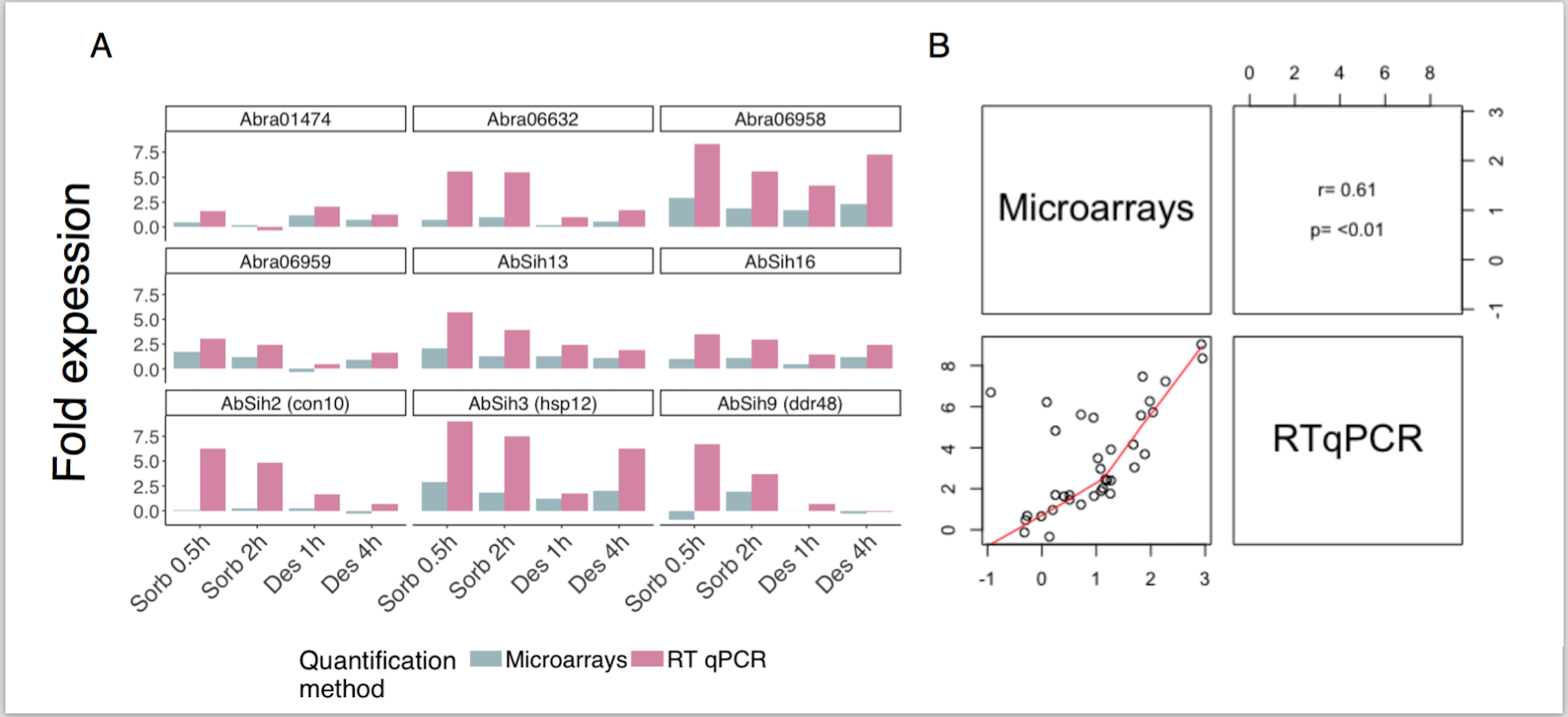

Supplement: FIGURE S1 — Comparison of transcript levels obtained by microarray or RT-qPCR methods. The expression of 9 genes was monitored in response to sorbitol and desiccation stresses. The fold inductions (log2 values) are presented in (A) and the correlation plot in (B). [file Image_1.TIFF]

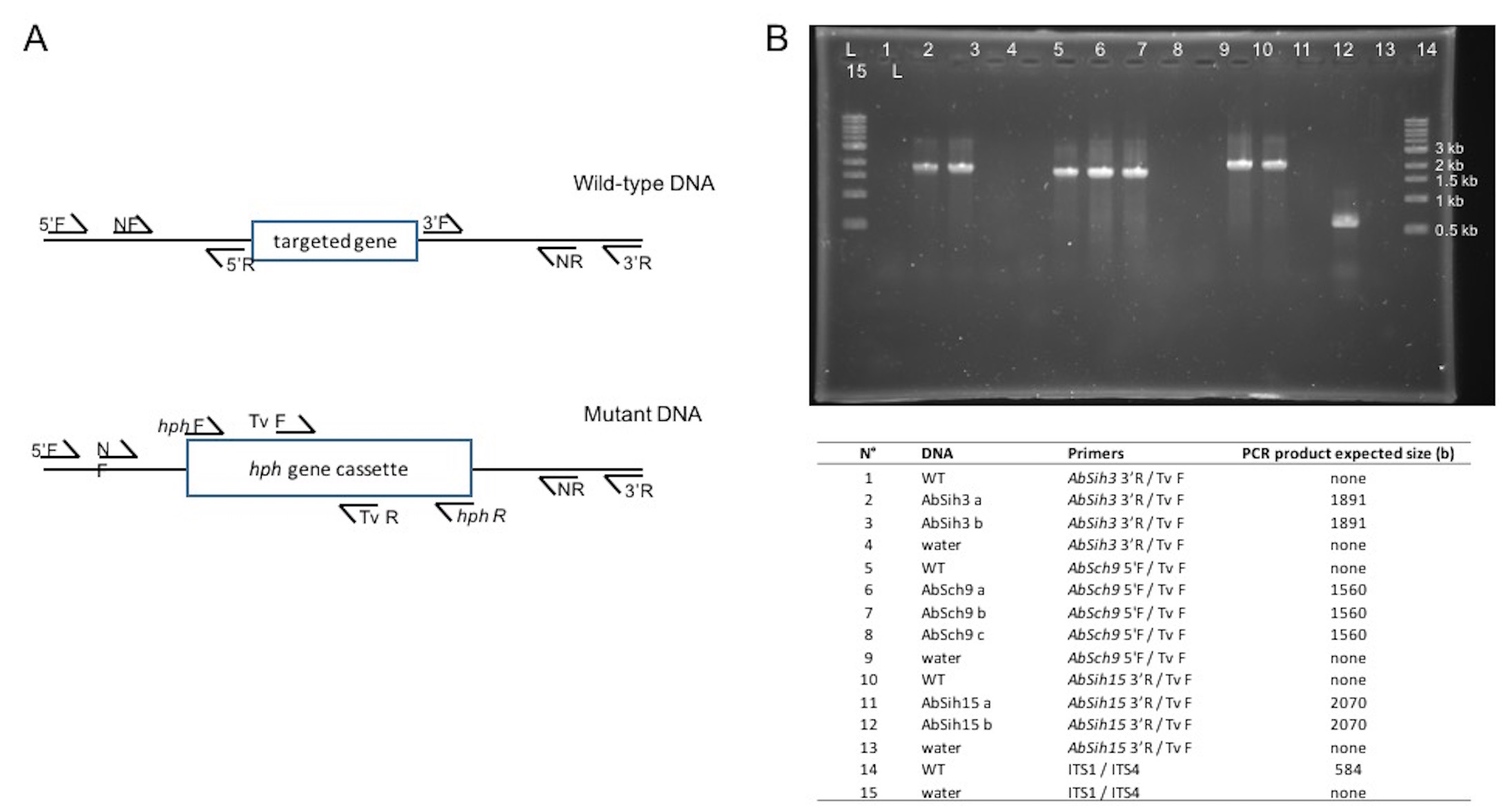

Supplement: FIGURE S3 — Generation of null mutants by homologous recombination. (A) Schematic representation of the deleted locus in the wild-type and the replacement construct with the Hyg B resistance cassette (Hph gene). Arrows indicate the position of primers used for the cassette generation and for PCR screening of mutants. (B) Gel electrophoresis of PCR products obtained from template DNA of the wild-type, Δabsch9, Δabsih3 and Δabsih15 strains with the indicated primer pairs. Molecular sizes (kb) were estimated based on a 1 kb ladder (lane M, NEW ENGLAND BioLabs® Inc.). A supplementary PCR was done with the primer pair ITS1/ITS4 as a positive control to assess the quality of the wild-type template DNA. [file Image_3.JPEG]
