# Supplementary material for: Responses to Hydric Stress in the Seed-Borne Necrotrophic Fungus Alternaria brassicicola
Source: Front Microbiol. 2019 Aug 30;10:1969. doi: 10.3389/fmicb.2019.01969 (PMC6730492; doi:10.3389/fmicb.2019.01969)
Supplement: TABLE S1 — List of predicted sequences from the Alternaria brassicicola genome exhibiting physicochemical features of hydrophilins and an obvious match upon Blast analysis with proteins of known functions. [file Table_1.DOCX]

| **Prot ID Ab43 genome** | **Prot ID ref genome** | **GRAVY** | **length** | **%G** | **%CC** | **Score** | **BLAST** |
| --- | --- | --- | --- | --- | --- | --- | --- |
| Abra00986 | AB01932 | -1,03 | 1025 | 8,1 | 66 | 4 | AIM 21 : subunit of a complex that associates with actin filaments |
| Abra1218 | AB01737 | -1,72 | 46 | 19,6 | 93 | 35675 | ubiquitine conjugating enzyme |
| Abra01627 | AB00980 | -1,09 | 264 | 9 | 52 | 18 | mitotic checkpoint protein |
| Abra05302 | AB01321 | -1,69 | 223 | 8,5 | 65 | 516 | splicing factor |
| Abra05386 | AB03443 | -1,14 | 542 | 10,1 | 59 | 255 | phosphodiesterase / Zn finger prot |
| Abra09139 | AB06279 | -1,38 | 1127 | 9,8 | 62 | 829 | splicing factor |
| Abra08664 | AB06667 | -1,29 | 291 | 12 | 52 | 235 | SAP like, DNA binding |
| Abra12180 | AB10150 | -1,25 | 238 | 17,6 | 57 | 1711 | glycosyltransferase |
| Abra00113 | AB02641 | -1,09 | 266 | 8,6 | 55 | 27 | RNA polymerase II subunit |
| Abra02972 | AB09472 | -1,12 | 831 | 10,2 | 65 | 390 | RNA-binding protein |
| Abra02978 | AB09472 | -1,02 | 112 | 10,7 | 84 | 221 | RNA-binding protein |
| Abra03188 | AB04539 | -1,10 | 388 | 26,8 | 71 | 4080 | mRNA splicing |
| Abra03583 | AB04233 | -1,49 | 243 | 8,2 | 58 | 78 | translation initiation factor |
| Abra04172 | AB03714 | -1,09 | 154 | 11,7 | 53 | 97 | IGO1 : initiation of the TORC1-regulated G0 program |
| Abra05339 | AB10536 | -1,07 | 618 | 12 | 65 | 437 | transcription elongation factor |
| Abra05505 | AB03352 | -1,05 | 861 | 9,1 | 53 | 17 | ARS2 : arsenite-resistance protein |
| Abra05514 | AB03343 | -1,21 | 245 | 10,2 | 67 | 772 | mRNA processing |
| Abra05559 | AB03301 | -1,08 | 179 | 8,4 | 61 | 34 | histone acetyltransferase |
| Abra05803 | AB03094 | -1,01 | 309 | 15,2 | 73 | 91 | RNA binding protein |
| Abra06090 | AB02851 | -1,09 | 476 | 13,4 | 57 | 330 | RNA binding protein |
| Abra06959 | AB03575 | -1,01 | 679 | 11,6 | 73 | 106 | protein kinase |
| Abra07818 | AB05183 | -1,32 | 862 | 8,5 | 58 | 127 | Rho GTPase |
| Abra07942 | AB05287/AB05286 | -1,06 | 573 | 9,8 | 56 | 68 | splicing factor |
| Abra08896 | AB06486 | -1,69 | 283 | 17,7 | 77 | 18157 | splicing factor |
| Abra09963 | AB07082 | -1,01 | 406 | 11,1 | 72 | 81 | glucanase |
| Abra10245 | AB09330 | -1,08 | 583 | 10,8 | 59 | 208 | protein kinase |
| Abra11865 | AB05724 | -1,54 | 462 | 10,2 | 58 | 948 | RNA binding protein |
| Abra01030 |  | -1,18 | 398 | 8,5 | 75 | 226 | transcription factor con7 |
| Abra03139 |  | -1,11 | 390 | 9,2 | 68 | 246 | IBD2 : component of the BUB2-dependent spindle checkpoint pathway |
| Abra07173 |  | -1,24 | 159 | 9,4 | 69 | 641 | ubiquinol cytochrome C reductase |
| Abra06168 | AB02776 | -1,04 | 102 | 11,8 | 66 | 232 | phosphodiesterase |
| Abra06505 | AB07550 | -1,21 | 96 | 9,4 | 55 | 147 | androgen receptor coactivator |
| Abra01481 | AB01113 | -1,26 | 107 | 8,4 | 87 | 386 | RNA binding protein |
| Abra00658 | AB02213 | -1,08 | 65 | 18,5 | 87 | 3048 | Protein involved in transport between Golgi and endosome |
| Abra00755 | AB02127 | -1,28 | 342 | 11,4 | 72 | 2084 | splicing factor |
| Abra00874 | AB02028 | -1,34 | 82 | 12,2 | 65 | 2136 | VanZ domain protein |
| Abra01413 | AB01168 | -1,06 | 168 | 10,1 | 58 | 96 | nuclear cap binding protein |
| Abra01458 | AB01134 | -1,03 | 638 | 8,2 | 66 | 10 | ribosomal protein |
| Abra01995 | AB00678 | -1,26 | 89 | 11,2 | 63 | 1084 | peptidyl-prolyl cis-trans isomerase |
| Abra02768 | AB00044 | -1,63 | 72 | 13,9 | 55 | 1848 | transport related protein |
| Abra03041 | AB09533 | -1,33 | 58 | 8,6 | 74 | 482 | zinc finger protein |
| Abra03628 | AB04189 | -1,19 | 539 | 11,5 | 72 | 1439 | translation initiation factor |
| Abra03849 | AB03980 | -1,51 | 112 | 8 | 71 | 0 | protein phosphatase |
| Abra04528 | AB08768 | -1,04 | 122 | 9 | 75 | 90 | ribosomal protein |
| Abra07200 | AB09078/AB03945 | -1,01 | 458 | 8,5 | 72 | 8 | transcription factor |
| Abra07202 | AB04602 | -1,12 | 598 | 8,9 | 59 | 99 | epsin |
| Abra08835 | AB06541 | -1,02 | 193 | 8,8 | 60 | 17 | hydrolase |
| Abra09386 | AB08475 | -1,09 | 171 | 9,4 | 66 | 206 | transcription factor like |
| Abra09470 | AB08399 | -1,13 | 107 | 11,2 | 72 | 934 | cytochrome C assembly |
| Abra10072 | AB07183 | -1,20 | 1079 | 9,3 | 64 | 368 | chitin synthase regulatory protein |
| Abra10114 | AB07217 | -1,00 | 380 | 10,3 | 67 | 2 | ribonuclease |
| Abra11573 | AB05968 | -1,14 | 285 | 15,4 | 81 | 3316 | GTPase activating GYP7 |
| Abra11784 | AB05788 | -1,22 | 35 | 11,4 | 85 | 2618 | translation initiation factor |
| Abra11876 | AB05716 | -1,01 | 52 | 9,6 | 71 | 26 | cyclin-like protein |
| Abra12345 | AB10014 | -1,07 |  | 17,3 | 58 | 521 | replication factor |
| Abra12386 | AB09980 | -1,14 |  | 11,4 | 78 | 1374 | histone deacetylation RXT3 |
| Abra12433 | AB09952 | -1,26 |  | 9,7 | 51 | 44 | ribosomal protein |
