# Supplementary material for: Responses to Hydric Stress in the Seed-Borne Necrotrophic Fungus Alternaria brassicicola
Source: Front Microbiol. 2019 Aug 30;10:1969. doi: 10.3389/fmicb.2019.01969 (PMC6730492; doi:10.3389/fmicb.2019.01969)
Supplement: TABLE S2 — List of primers used in this study. [file Table_2.DOCX]

| **Genes** | **Use** | **Primers** |
| --- | --- | --- |
| *AbSih3* | 5' flanking regions for K.O | F :GCCCTCCTGTTAATGTCCCT |
|  |  | R :GTCGTGACTGGGAAAACCCTGGCGTGTTGACGGTTTGTTGGGTC |
|  | 3' flanking regions for K.O | F :TCCTGTGTGAAATTGTTATCCGCTCACCGGCACTCACACTACTC |
|  |  | R :GTCACGGAGGAATCTGAAGAG |
|  | Nested for K.O | F :GTATCCTGACTGTTGTGCCA |
|  |  | R :GTGGGTCTAAAGCCTATGGATCTG |
|  | Real time PCR | F :AGCCCGACTCTTCAAAAACC |
|  |  | R :AGGGCGTTTTTGGTCTTGTC |
| *AbSch9* | 5' flanking regions for K.O | F :ATAGAGGACGGAGAGATTGGTATTT |
|  |  | R :TCCTGTGTGAAATTGTTATCCGCTTATCAATATCTACCAACGTGATTGC |
|  | 3' flanking regions for K.O | F :GTCGTGACTGGGAAAACCCTGGCGTCAAGTTCACTTGTTTATGCATGTT |
|  |  | R :TTCCCACACAAAAGTACAGACTACA |
|  | Nested for K.O | F :AAGGTGAATGTACTACGGATGGTTA |
|  |  | R :GAGATATTGCATGTTATCCCAAGTC |
| *AbSih15* | 5' flanking regions for K.O | F :CCTGGTGGTTCAACGGTAAC |
|  |  | R :GTCGTGACTGGGAAAACCCTGGCGGAGTCGTGGATTGTGGCTTC |
|  | 3' flanking regions for K.O | F :TCCTGTGTGAAATTGTTATCCGCTCACGTCATATGCAGCGGTAG |
|  |  | R :AACGAGAAGAATGGGGAGGG |
|  | Nested for K.O | F :GAGCTCCGCCGTAAACAAAT |
|  |  | R :CCTGCTTGCTTCTTTCGTGT |
| *hph* | *hph* amplification | F :CGTTGCAAGACCTGCCTGAA |
|  |  | R :GGATGCCTCCGCTCGAAGTA |
|  | Transformant validation | F :CGTTGCAAGACCTGCCTGAA |
|  |  | R :GGATGCCTCCGCTCGAAGTA |
| *AB01474* | Real time PCR | F :GTGCAGAAGAAGGAGGTTGC |
|  |  | R :GTTGCAAGGCTAGGATGAGC |
| *AB02043* | Real time PCR | F :AGTCCACATTGTCGGCTACC |
|  |  | R :CCATATCGCTAGGCAAGGAG |
| *AB06632* | Real time PCR | F :CAAGCTAGCGCAGATCCTCT |
|  |  | R :TAAGCTTCGATTGCCTGGTT |
| *AB06958* | Real time PCR | F :AGCTTGGTCCCAATGTCAAG |
|  |  | R :TCATCGGGAACCTTGAAGAC |
| *AB01782 (con10)* | Real time PCR | F :GGCTTCATCCGGTAGTTTTG |
|  |  | R :AGACGATTGTCCACCTTTCC |
| *AB02794 / AbSih13* | Real time PCR | F :CCGGAAACGTTTACAAGGAG |
|  |  | R :AAAGGGGAAGGCTCATGTTC |
| *AB03330 / AbSih9* | Real time PCR | F :CCTACGGCAACAAAAAGTCC |
|  |  | R :CGATGACCCAAGAATGGAAG |
| *AB05433 / AbSih16* | Real time PCR | F :AAGACAAATGGGACGAGCAC |
|  |  | R :TGTTGCTGTCCGTACTTTCG |
| *AB05812 (adenylyl cyclase-associated protein)* | Real time PCR | F :GAAGAAGCCACCGAAGAAG |
|  |  | R :GAGATGAGAATGGAGTGGTTG |
| AB07630 (prefolding subunit 1) | Real time PCR | F :GCAGAAGAGACTCAAGGATG |
|  |  | R :CGAGGCTGTTCTTGCTATT |
| AB07628 (β-tubuline) | Real time PCR | F :TTCAACGAAGCCTCCAACAAC |
|  |  | R :GTGCCGGGCTCGAGAT |
| ITS | Wil type DNA template validation | F :TCCGTAGGTGAACCTGCGG |
|  |  | R :TCCTCCGCTTATTGATATGC |
